# Coexpression of IQ-Domain GTPase-Activating Protein 1 (IQGAP1) and Dishevelled (Dvl) Is Correlated with Poor Prognosis in Non-Small Cell Lung Cancer

**DOI:** 10.1371/journal.pone.0113713

**Published:** 2014-12-01

**Authors:** Huanyu Zhao, Chengyao Xie, Xuyong Lin, Yue Zhao, Yang Han, Chuifeng Fan, Xiupeng Zhang, Jiang Du, Yong Han, Qiang Han, Guangping Wu, Enhua Wang

**Affiliations:** Department of Pathology, First Affiliated Hospital and College of Basic Medical Sciences, China Medical University, Shenyang, China; Univesity of Texas Southwestern Medical Center at Dallas, United States of America

## Abstract

**Background:**

IQ-domain GTPase-activating protein 1 (IQGAP1) binds to Dishevelled (Dvl) and functions as a modulator of Dvl nuclear localization in Xenopus embryos. However, the relationship between IQGAP1 and Dvl in tumor tissues is unclear.

**Materials and Methods:**

We used immunohistochemistry to assess the expressions of IQGAP1 and Dvl in a cohort of 111 non-small cell lung cancer (NSCLC) patients. Association of their localization expressions with clinicopathological factors was also analyzed.

**Results:**

The positive rate of IQGAP1 in primary tumors was 48.6% (54/111) for its cytoplamic expression, 9.0% (10/111) for nuclear expression and 31.5% (35/111) for membranous expression; the positive rate of Dvl was 65.8% (73/111) for cytoplamic expression, 9.9% (11/111) for nuclear expression and 10.8% (12/111) for membranous expression. Coexpression rate of IQGAP1 and Dvl was 77.8% (42/54) in the cytoplasm, 80.0% (8/10) in the nucleus and 8.6% (3/35) in the membrane. Coexpression of IQGAP1 and Dvl in the cytoplasm and nucleus were significantly correlated (P<0.05), but not in the membrane (P>0.05). The positive expression rates of cyclin D1 and c-myc were significantly higher in the group of IQGAP1 and Dvl coexpression in the nucleus than that in the cytoplasm. Coexpression rate of IQGAP1 and Dvl in the cytoplasm and nucleus was significantly higher in lymph nodal metastases (63.3%, 19/30) than in primary growths (38.3%, 31/81), correlating with poor prognosis. Five-year survival time after resection in the group with their coexpression in the cytoplasm and nucleus was significantly lower than that with no coexpression (44.705±3.355 vs 58.403±2.543 months, p<0.05).

**Conclusions:**

Coexpression of IQGAP1 and Dvl in the cytoplasm and nucleus was correlated with the lymph nodal metastase and poor prognosis of NSCLC, and coexpression in nucleus might play a critical role in the activation of canonical Wnt pathway.

## Introduction

IQGAP family proteins are found in numerous organisms, including yeast, fish, and mammals. There are three isoforms of IQGAP in humans: IQGAP1, IQGAP2 and IQGAP3. IQGAP1 is the best characterized and the most widely studied member of the IQGAP family. It is a scaffolding protein that binds to filamentous actin and functions to cross-link and stabilize actin filaments via its calponin homology (CH) domain at the N-terminus [1]. Previous reports showed that IQGAP1 influenced cell motility at the leading edge of migrating cells, by increasing the levels of active Rac1 and Cdc42 [2]. IQGAP1 shows elevated levels in a variety of cancer types, including pancreatic cancer [3]. The expression and subcellular location of IQGAP1 in lung adenocarcinoma was associated with histologic differentiation and can be used to predict survival in patients [4]. Recent research suggests that IQGAP1 is a risk factor for lymph node metastasis of lung squamous cell carcinomas [5].

Dvl has been identified as a key regulator of Wnt signaling (including canonical and noncanonical pathways),which is a key component of physiological process involved in embryonic development and tumor progression [6], [7]. Many reports have demonstrated the role of Dvl in tumors. Dvl overexpression is significantly correlated with poor differentiation and lymph node metastasis in NSCLC [8]. Dvl-1 and Dvl-3 affect NSCLC cell invasion mainly through canonical and noncanonical Wnt pathways, respectively [9]. Wnt5a promotes breast cancer cell migration via Dvl-2 [10].

IQGAP1 functions as a modulator of Dvl nuclear localization in Wnt signaling [11]. However, the correlation between IQGAP1 and Dvl in tumors is unclear. In the present study, we performed an immunohistochemical analysis to identify the expressions and locations of IQGAP1 and Dvl in NSCLC. Moreover, we analyzed their association with clinicopathological parameters.

## Materials and Methods

### Ethics Statement

All human tissues were obtained in accordance with Human Subject Research Protocols approved by the China Medical University Review Board. Tumor tissues were obtained with written informed consent from adult patients with NSCLC.

### Tissue samples and patient data

We collected 111 specimens from NSCLC patients who underwent complete resection in the First Affiliated Hospital of China Medical University from January to December of 2008. None of the patients had received radiotherapy or chemotherapy before surgical resection. Follow-up information was obtained from review of the patients' medical record. The pTNM staging system of the International Union Against Cancer (7th edition) was used in our study. This study was conducted with the approval of the local institutional review board at China Medical University. The main clinical and pathological variables of all patients are as follow: 46 cases of squamous cell carcinoma, 65 cases of adenocarcinoma; 38 cases of well differentiation, 73 cases of moderate-poor differentiation; 67 cases in stage I, 20 cases in stage II, 24 cases were stage III; 70 cases of male patients, 41 cases of female patients; the average age is 57 years old.

### Immunohistochemistry

Surgically excised tumor specimens were fixed with 10% neutral formalin, embedded in paraffin and 4 µm thick sections were prepared. Immunohistochemical staining was performed using the avidin–biotin–peroxidase complex method (UltrasensitiveTM, MaiXin, Fuzhou, China). The sections were deparaffinized in xylene, rehydrated with graded alcohol, and then boiled in 0.01 M citrate buffer (pH 6.0) for 2 min with an autoclave. Hydrogen peroxide (0.3%) was applied to block endogenous peroxide activity and the sections were incubated with normal goat serum to reduce non-specific binding. Tissue sections were then incubated with primary antibodies overnight at 4°C. Primary antibodies (mouse anti-human) are as follows: IQGAP1 monoclonal antibody (1∶40, sc-376021, Santa Cruz Biotechnology, USA), Dvl monoclonal antibody (1∶25, sc-166303, Santa Cruz), cyclin D1 monoclonal antibody (1∶100, sc-20044, Santa Cruz), c-myc monoclonal antibody (1∶500, cat.ab32, Abcam). Biotinylated goat antimouse serum IgG was used as a secondary antibody. As an isotype control, mouse IgG (Maixin Biotechnology, Fuzhou, Fujian, China, at the same concentration of the primary antibody) was used instead of the primary antibody (Figure S1). After washing, the sections were incubated with streptavidin-biotin conjugated with horseradish peroxidase, and the peroxidase reaction was developed with 3,3′-diaminobenzidine tetrahydrochloride. Counterstaining was done with hematoxylin, and the sections were then dehydrated with ethanol before being mounted.

### Evaluation of staining

The staining intensity and percentage of cells stained in representative areas of each slide were independently evaluated and scored by two investigators, who were blinded to the clinical data about patients. Five views were examined per slide, and 100 cells were observed per view at 400× magnification. For IQGAP1 or Dvl, the immunohistochemical results showed that positive staining localized in the nucleus, cytoplasm, and (or) membrane. Tumors were defined as cytoplasmic expression when 20% or more tumor cells showed cytoplasmic staining; tumors were defined as nuclear expression when 20% or more tumor cells showed nuclear staining,whether cytoplasmic staining or not; tumors were defined as membranous expression when 20% or more tumor cells showed membranous staining, whether cytoplasmic staining or not. The regions with positive expression were yellow or brown-yellow, and the regions with negative expression were no staining. For cyclin D1 or c-myc, the immunohistochemical results showed that positive staining localized in the nucleus or nucleus/cytoplasm.

### Statistical analysis

SPSS version 16.0 for Windows was used for all analyses. The Chi-squared test was used to examine various possible correlations. Kaplan–Meier survival analysis was used to estimate the probability of patients' survival, and the differences of survival between the subgroups of patients. The Cox proportional hazard regression model was used to estimate the possible prognostic significance of clinicopathological variables. All P values were based on the two-sided statistical analysis, and P<0.05 was considered to be statistically significant in difference.

## Results

### 1. Coexpression of IQGAP1 and Dvl mainly in the cytoplasm and nucleus

The positive expression rate of IQGAP1 was 89.2% (99/111), and 48.6% (54/111) for its cytoplamic expression, 9.0% (10/111) for nuclear expression and 31.5% (35/111) for membranous expression. The positive expression rate of Dvl was 86.5% (96/111), and 65.8% (73/111) for cytoplamic expression, 9.9% (11/111) for nuclear expression and 10.8% (12/111) for membranous expression (Table S1).

**Figure 1:**
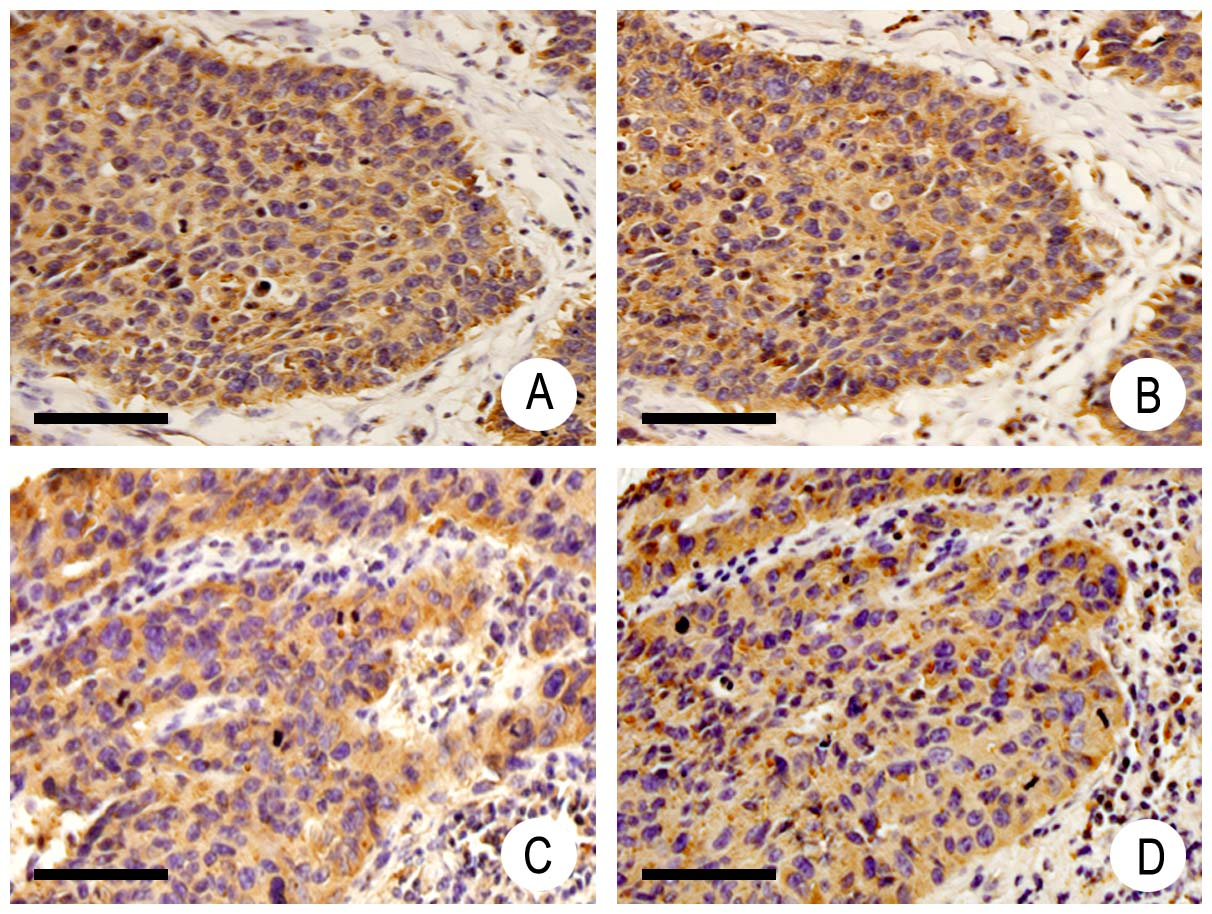
Coexpression of IQGAP1 (A, C) and Dvl (B, D) in the cytoplasm of NSCLC. A–B: adenocarcinoma; C–D: squamous cell carcinoma. Original magnification, 400×; scale bar, 20 µm.

**Figure 2:**
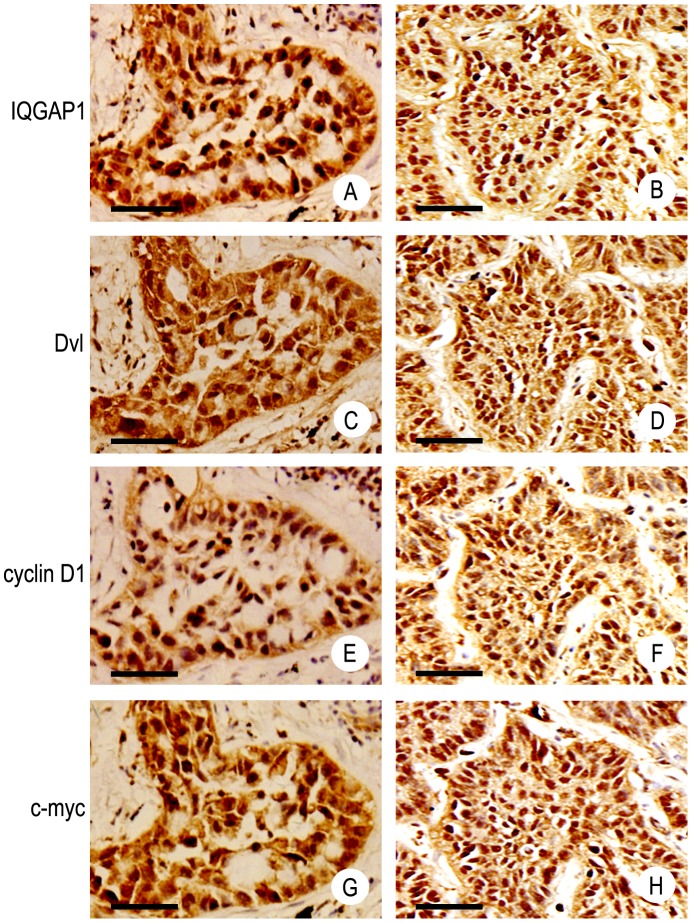
Nuclear coexpression of IQGAP1 and Dvl is correlated with cyclin D1 and c-myc expression. Coexpression of IQGAP1 (A) and Dvl (C) in the nucleus of lung adenocarcinoma. Coexpression of IQGAP1 (B) and Dvl (D) in the nucleus of lung squamous cell carcinoma. Positive expression of cyclin D1 (E) and c-myc (G) in lung adenocarcinoma. Positive expression of cyclin D1 (F) and c-myc (H) in lung squamous cell carcinoma. Original magnification, 400×; scale bar, 20 µm.

Coexpression rate of IQGAP1 and Dvl was 77.8% (42/54) in the cytoplasm (Table 1, Figure 1), 80.0% (8/10) in the nucleus (Table 1, Figure 2A–D). Coexpression in the cytoplasm was significantly correlated (Table 1, P = 0.009, r = 0.246), also in the nucleus (Table 1, P = 0.000, r = 0.738); coexpression rate of IQGAP1 and Dvl in the membrane was only 8.6% (3/35), and coexpression in the membrane was not correlated (Table 1, P>0.05, Figure S2).

**Table 1:** The relationship between IQGAP1 and Dvl expression in NSCLC.

	IQGAP1								
Dvl	Cyt-coexpression			Nuc-coexpression			Mem-coexpression		
	No	Yes	Total	No	Yes	Total	No	Yes	Total
No	26	12	38	98	2	100	67	32	99
Yes	31	42	73	3	8	11	9	3	12
Total	57	54	111	101	10	111	76	35	111

Cyt: cytoplamic; Nuc: nuclear; Mem: membranous.

The expression of cyclin D1 and c-myc was significantly positive in the group of IQGAP1 and Dvl coexpression in the nucleus (Figure 2E–H), and their positive expression rates were 100% (8/8) and 87.5% (7/8), respectively. In the group of IQGAP1 and Dvl coexpression in the cytoplasm, the positive expression rates of cyclin D1 and c-myc were 52.4% (22/42) and 35.7% (15/42), respectively. The positive expression rates of cyclin D1 and c-myc in the group of nuclear coexpression were significantly higher than that of cytoplasmic coexpression (P<0.05).

### 2. Coexpression of IQGAP1 and Dvl in the cytoplasm or nucleus was correlated to lymphatic metastasis of NSCLC

As shown in Table 2, coexpression rate of IQGAP1 and Dvl in the cytoplasm and nucleus was 63.3% (19/30) in the group with lymph nodal metastases (N1-3), and 38.3% (31/81) in the group with primary growths (N0). Nodal metastases showed higher coexpression rate of IQGAP1 and Dvl in the cytoplasm and nucleus than primary growths (P<0.05). No statistically significant different rate was shown between the group of T1 and T2-4 (tumor status) (P>0.05, Table S2), also between the group of adenocarcinoma and squamous cell carcinoma (histological types) (P>0.05, Table S3).

**Table 2:** IQGAP1 and Dvl coexpression in different N stage.

	IQGAP1							
Dvl	N0				N1-3			
	Neg	Cyt	Nuc	Mem	Neg	Cyt	Nuc	Mem
Neg	0	7	1	4	0	2	1	0
Cyt	2	25	0	23	1	17	0	5
Nuc	0	3	6	0	0	0	2	0
Mem	8	0	0	2	1	0	0	1
Total	10	35	7	29	2	19	3	6

Neg: negative; Cyt: cytoplasm; Nuc: nuclear; Mem: membrane.

### 3. Coexpression of IQGAP1 and Dvl in the cytoplasm or nucleus was correlated to poor prognosis of NSCLC

**Figure 3:**
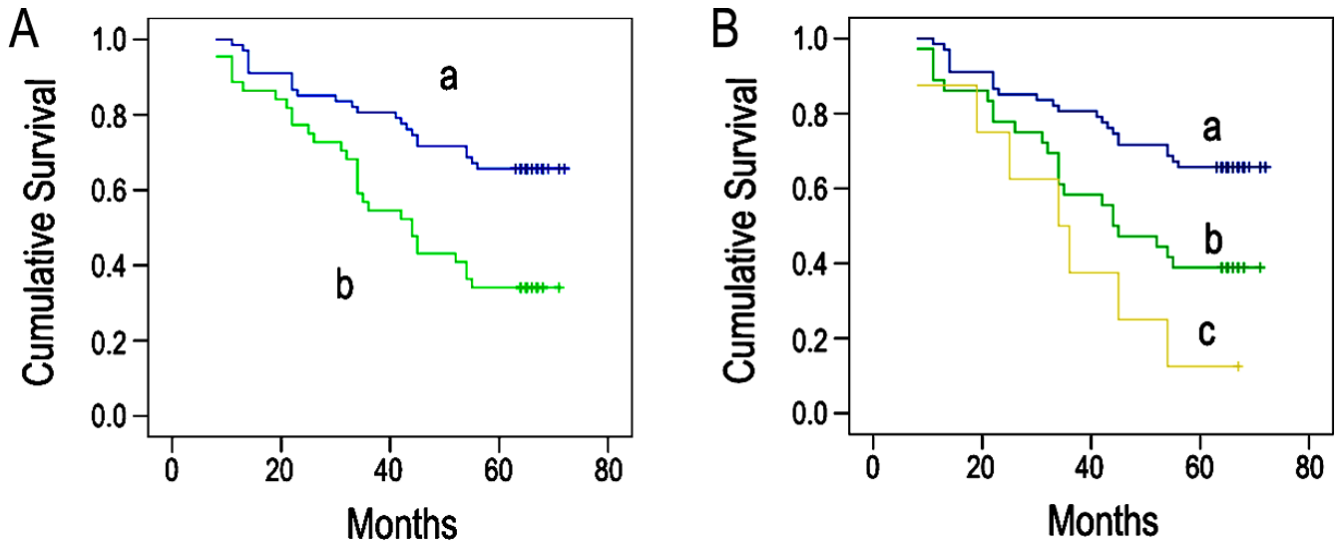
Kaplan–Meier analysis showing overall survival among NSCLC patients. (A) a.the group with IQGAP1 and Dvl noncoexpression in the cytoplasm and nucleus (61 cases); b.the group with IQGAP1 and Dvl coexpression in the cytoplasm and nucleus (50 cases) (B) a.the group with IQGAP1 and Dvl noncoexpression in the cytoplasm and nucleus (61 cases); b.the group with IQGAP1 and Dvl coexpression in the cytoplasm (42 cases); c.the group with IQGAP1 and Dvl coexpression in the nucleus (8 cases).

Kaplan–Meier survival analysis showed a significantly lower survival time in patients with coexpression of IQGAP1 and Dvl in the cytoplasm and nucleus than those with no coexpression. Five-year survival time after resection in the group with their coexpression in the cytoplasm and nucleus was significantly lower than that with no coexpression (44.705±3.355 vs 58.403±2.543 months, p<0.05, Figure 3A), and the group of nuclear coexpression was significantly lower than that of cytoplasmic coexpression (36.000±6.329 vs 46.528±3.758 months, P<0.05, Figure 3B).

To clarify whether coexpression of IQGAP1 and Dvl in the cytoplasm and nucleus was independently associated with the prognosis of patients with NSCLC, we employed the Cox proportional hazard regression model. The result showed that cytoplasmic and nuclear coexpressions were independent risk factors for the prognosis of patients with NSCLC (P<0.05; Table 3).

**Table 3:** Cox Regression Model for Prediction of 111 Patients With Lung Cancer.

Factor	P-value	95% Cl	Risk
Gender	0.929	0.508–1.856	0.971
Differentiation	0.510	0.270–0.962	0.038
Histology	0.657	0.626–2.102	1.147
pTNM stage	0.028	0.242–0.776	0.433
Lymph node metastasis	0.005	1.087–4.453	2.200
Cytoplasmic coexpression of IQGAP1 and Dvl	0.031	0.211–0.799	0.455
Nuclear coexpression of IQGAP1 and Dvl	0.020	0.374–1.403	0.615

## Discussion

As an important effector of Rho GTPases, IQGAP1 is a scaffold protein and plays foundational function in cell movement by modulating the actin cytoskeleton through Rac1 and Cdc42 [12]–[14]. Dvl is an essential mediator of the canonical Wnt signaling pathway, and its nuclear localization is required for canonical Wnt signaling [15], [16]. In Xenopus embryos,IQGAP1 interacted with Dvl and regulated its nuclear localization to moderate the expression of Wnt target genes during early embryogenesis [11]. However,the relationship between IQGAP1 and Dvl in tumors has not been reported.

The present study investigated the IQGAP1 and Dvl expression of NSCLC (adenocarcinoma and squamous cell carcinoma). We found that IQGAP1 and Dvl were mainly located in the cytoplasm. Coexpression rate of IQGAP1 and Dvl in the cytoplasm or nucleus was significantly higher than that in the membrane; coexpression of IQGAP1 and Dvl in the cytoplasm and nucleus were significantly correlated (P<0.05), but not in the membrane (P>0.05). The coexpression of IQGAP1 and Dvl in the cytoplasm and nucleus was associated with lymph node metastasis and poor prognosis in NSCLC. The survival in the group of nuclear coexpression was significantly worse than that of cytoplasmic coexpression. The membranous coexpression of IQGAP1 and Dvl were not correlated (P>0.05). These results suggested that IQGAP1 interacted with Dvl in the cytoplasm and nucleus of NSCLC, and the role in the cytoplasm might be one of the critical steps in modulating Dvl nuclear localization, but not in the membrane. These findings are consistent with previous study [11].

Nuclear localization of Dvl is required for canonical Wnt signaling [15]. Dvl cooperates with c-Jun to regulate gene transcription stimulated by the canonical Wnt signaling pathway in the nucleus [16]. In this study, we found that the target genes of canonical Wnt signaling, cyclin D1 and c-myc, the positive rates of them in the group with IQGAP1 and Dvl coexpression in the nucleus were much higher than that in the cytoplasm. Survival analysis also showed that patients with coexpression of IQGAP1 and Dvl in the nucleus had a significantly lower survival time than patients with that in the cytoplasm (P<0.05). These results suggest that nuclear coexpression of IQGAP1 and Dvl is associated with the activation of canonical Wnt pathway.

Based on the above results we conclude that coexpression of IQGAP1 and Dvl in the cytoplasm and nucleus is correlated with lymph node metastasis and poor prognosis in NSCLC. IQGAP1 modulates Dvl nuclear localization in the cytoplasm, but not in the membrane. The coexpression of IQGAP1 and Dvl in the nucleus is associated with the expression of cyclin D1 and c-myc, which suggest that nuclear coexpression of IQGAP1 and Dvl is associated with the activation of canonical Wnt pathway. Therefore, inhibition of the binding of IQGAP1 and Dvl in the cytoplasm and preventing Dvl nuclear translocation, might be one of the strategies for the prevention and treatment of lung cancer.

## Supporting Information

Figure S1 **The validation of antibody (IQGAP1, A and D; Dvl, B and E) for immunohistochemistry.** A–C: adenocarcinoma; D–F: squamous cell carcinoma; C and F: mouse IgG was used instead of the primary antibody. Original magnification, 400×; scale bar, 20 µm.
(TIF)

Figure S2 **Coexpression of IQGAP1 (A, C) and Dvl (B, D) in the membrane of NSCLC.** A–B: adenocarcinoma; C–D: squamous cell carcinoma. Original magnification, 400×; scale bar, 20 µm.
(TIF)

Table S1 **IQGAP1 and Dvl expression in NSCLC.**
(DOC)

Table S2 **IQGAP1 and Dvl coexpression in different tumor status.**
(DOC)

Table S3 **IQGAP1 and Dvl coexpression in different histological types.**
(DOC)
