# Supplementary figures and images for: Coexpression of IQ-Domain GTPase-Activating Protein 1 (IQGAP1) and Dishevelled (Dvl) Is Correlated with Poor Prognosis in Non-Small Cell Lung Cancer
Source: PLoS One. 2014 Dec 1;9(12):e113713. doi: 10.1371/journal.pone.0113713 (PMC4249885; doi:10.1371/journal.pone.0113713)

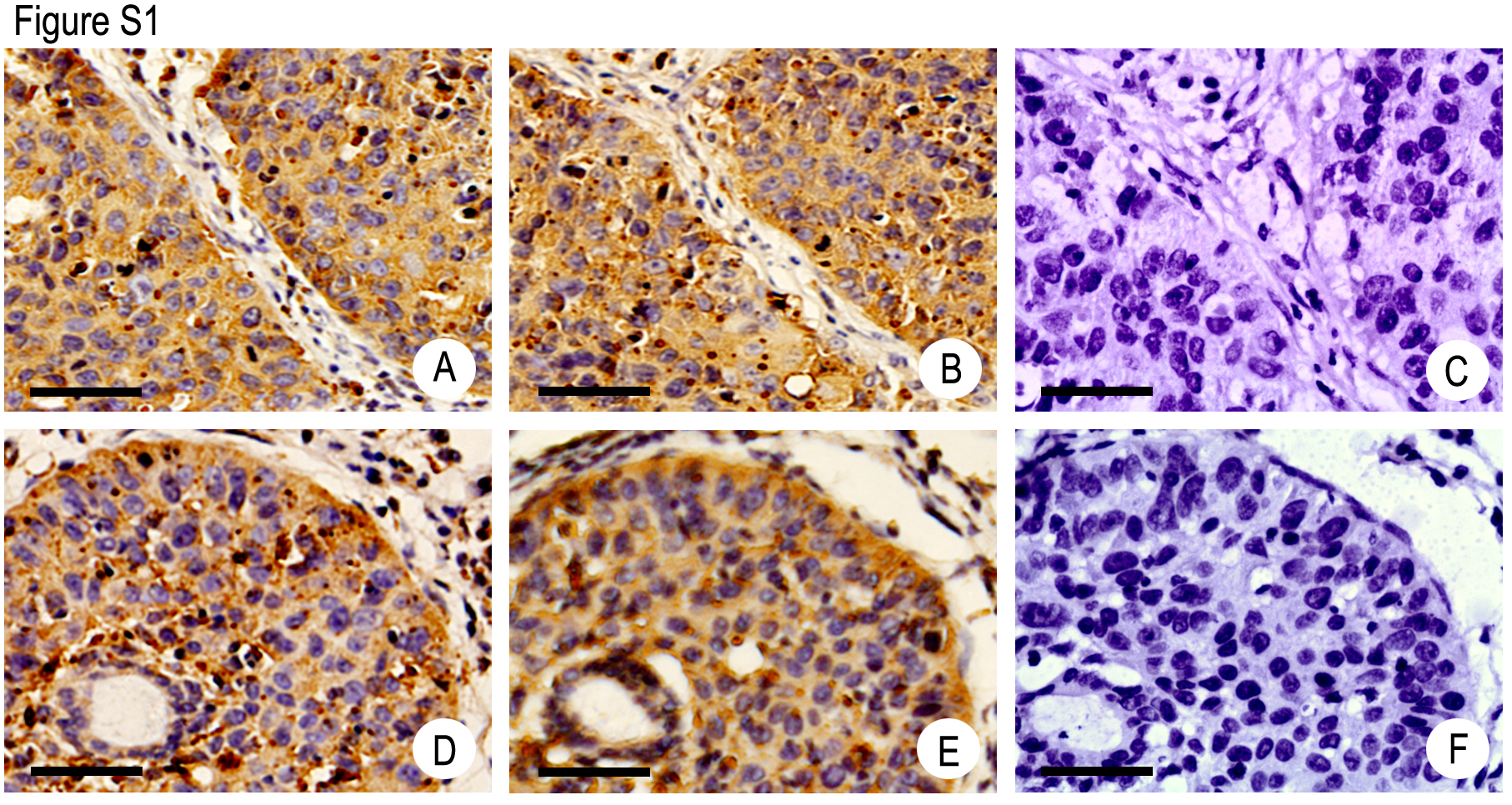

Supplement: Figure S1 — The validation of antibody (IQGAP1, A and D; Dvl, B and E) for immunohistochemistry. A–C: adenocarcinoma; D–F: squamous cell carcinoma; C and F: mouse IgG was used instead of the primary antibody. Original magnification, 400×; scale bar, 20 µm. (TIF) [file pone.0113713.s001.tif]

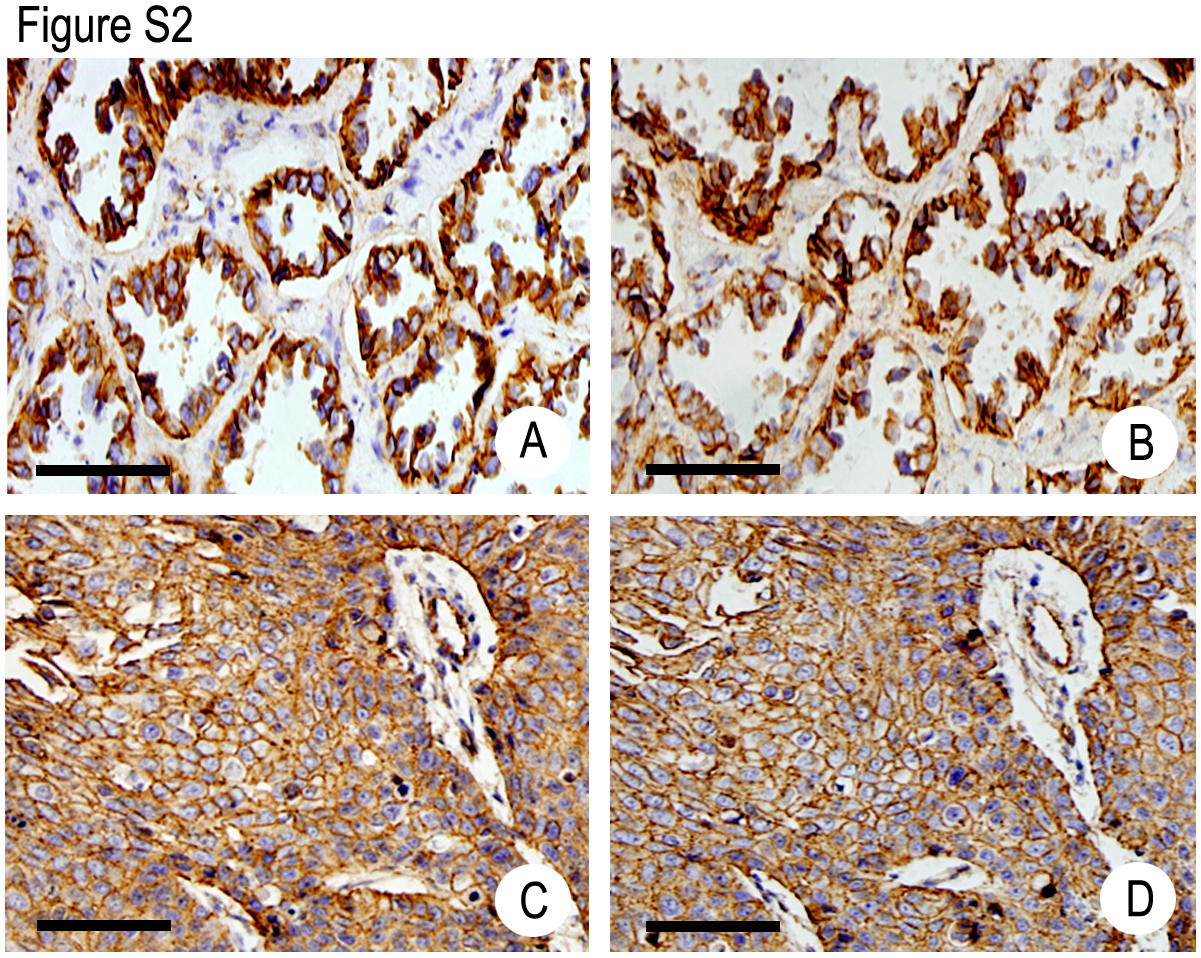

Supplement: Figure S2 — Coexpression of IQGAP1 (A, C) and Dvl (B, D) in the membrane of NSCLC. A–B: adenocarcinoma; C–D: squamous cell carcinoma. Original magnification, 400×; scale bar, 20 µm. (TIF) [file pone.0113713.s002.tif]
