# Supplementary material for: Coexpression of IQ-Domain GTPase-Activating Protein 1 (IQGAP1) and Dishevelled (Dvl) Is Correlated with Poor Prognosis in Non-Small Cell Lung Cancer
Source: PLoS One. 2014 Dec 1;9(12):e113713. doi: 10.1371/journal.pone.0113713 (PMC4249885; doi:10.1371/journal.pone.0113713)
Supplement: Table S1 — IQGAP1 and Dvl expression in NSCLC. (DOC) [file pone.0113713.s003.doc]

| Table S1. IQGAP1 and Dvl expression in NSCLC | | | | | |
| --- | --- | --- | --- | --- | --- |
| Dvl | IQGAP1 | | | | |
|  | Neg | Cyt | Nuc | Mem | Total |
| Neg | 0 | 9 | 2 | 4 | 15 |
| Cyt | 3 | 42 | 0 | 28 | 73 |
| Nuc | 0 | 3 | 8 | 0 | 11 |
| Mem | 9 | 0 | 0 | 3 | 12 |
| Total | 12 | 54 | 10 | 35 | 111 |
| Neg: negative; Cyt: cytoplasm;  Nuc: nuclear; Mem: membrane | | | | | |
