# Supplementary material for: Coexpression of IQ-Domain GTPase-Activating Protein 1 (IQGAP1) and Dishevelled (Dvl) Is Correlated with Poor Prognosis in Non-Small Cell Lung Cancer
Source: PLoS One. 2014 Dec 1;9(12):e113713. doi: 10.1371/journal.pone.0113713 (PMC4249885; doi:10.1371/journal.pone.0113713)
Supplement: Table S2 — IQGAP1 and Dvl coexpression in different tumor status. (DOC) [file pone.0113713.s004.doc]

| Table S2. IQGAP1 and Dvl coexpression in different tumor status | | | | | | | | |
| --- | --- | --- | --- | --- | --- | --- | --- | --- |
|  | IQGAP1 | | | | | | | |
| Dvl | T1 | | | | T2-4 | | | |
|  | Neg | Cyt | Nuc | Mem | Neg | Cyt | Nuc | Mem |
| Neg | 0 | 2 | 1 | 2 | 0 | 7 | 1 | 2 |
| Cyt | 1 | 10 | 0 | 7 | 2 | 32 | 0 | 21 |
| Nuc | 0 | 1 | 5 | 0 | 0 | 2 | 3 | 0 |
| Mem | 2 | 0 | 0 | 3 | 7 | 0 | 0 | 0 |
| Total | 3 | 13 | 6 | 12 | 9 | 41 | 4 | 23 |
| Neg: negative; Cyt: cytoplasm; Mem: membrane; Nuc:nuclear | | | | | | | | |
